# Early Intracranial Hemorrhage Predicts Poor Clinical Outcome in Community-Acquired Bacterial Meningitis

**DOI:** 10.3389/fneur.2022.869716

**Published:** 2022-06-16

**Authors:** Johannes Weller, Jonas Simon Enkirch, Felix Lehmann, Alexander Radbruch, Thomas Klockgether, Julian Zimmermann

**Affiliations:** ^1^Department of Neurology, University Hospital Bonn, Bonn, Germany; ^2^Department of Neuroradiology, University Hospital Bonn, Bonn, Germany; ^3^Department of Anesthesiology, University Hospital Bonn, Bonn, Germany

**Keywords:** meningitis, central nervous system infection, bacterial meningitis, cerebral hemorrhage, intracranial hemorrhage, clinical outcome

## Abstract

**Background:**

Community-acquired bacterial meningitis in adults is associated with significant morbidity and mortality; therefore, early prognostication is important to identify severe cases and possibly allocate more intensive treatment. We hypothesized that early intracranial hemorrhage portends a poor prognosis. The objective of this study was to evaluate the prognostic impact of early intracranial hemorrhage regardless of size and location on clinical outcome.

**Methods:**

Retrospective analysis of patients with community-acquired bacterial meningitis treated at a tertiary academic center between 2009 and 2019 about patient characteristics, cerebral imaging findings, and clinical outcome. Uni- and multivariable logistic regression analyses were performed to identify independent predictors of poor clinical outcomes defined as a modified Rankin scale score of 5 or 6 upon discharge.

**Results:**

A total of 102 patients were included, of which 22.5% had poor clinical outcomes. Intracranial micro- or macrohemorrhages were present in 7.8% of cases and associated with poor clinical outcomes [odds ratio (OR) 55.75, 95% CI 3.08–1,008.48, *p* = 0.006] in multivariate analysis, further predictors included ischemic stroke (OR 15.06, 95% CI 1.32–172, *p* = 0.029), age (OR 2.56, 95% CI 1.4–4.67, *p* = 0.002), and reduced consciousness (OR 4.21, 95% CI 1.07–16.64, *p* = 0.04).

**Conclusion:**

Early cerebral hemorrhage (ECHO) is a potential prognostic marker for clinicians confronted with decision-making in patients who are critically ill with community-acquired bacterial meningitis.

## Introduction

Community-acquired bacterial meningitis is an infectious disease with high rates of morbidity and mortality (1). In clinical practice, prognostication of neurological outcomes in meningitis is difficult. On one hand, patients who are seemingly hopeless may survive and fully recover (2). On the other hand, deleterious complications such as stroke, brain edema, or hydrocephalus may occur and worsen outcomes (3). Large prospective studies set out to identify prognostic factors (4–6). As expected, advanced age, signs of more severe neurological affection, and systemic inflammation were prognostic for unfavorable outcome (3). Other factors remained equivocal, e.g., both absence and presence of otitis were associated with an unfavorable outcome (4, 6). While some information is available upon diagnosis, other prognostic factors, such as a positive blood culture or the causative pathogen, are available only during the course of disease (6).

The retrospective analyses reported high rates of cerebrovascular complications in meningitis negatively impacting outcomes, including intracerebral hemorrhage in 2–9% (7–10). While the association of territorial infarction or large intracranial hemorrhage with poor outcome comes as no surprise, an autopsy study was able to identify cerebral microhemorrhages in 67% of deceased patients with pneumococcal meningitis (11). Based on these findings, we hypothesized that intracranial hemorrhagic lesions, regardless of size, might be of prognostic value in bacterial meningitis, portending a poor prognosis and thus improving prognostication upon diagnosis or early in the course of the disease. Therefore, we performed an analysis of patients with community-acquired bacterial meningitis to evaluate the presence of early cerebral hemorrhage (ECHO) and its impact on clinical outcomes.

## Materials and Methods

### Study Design and Patient Characteristics

We retrospectively included all adult patients (age ≥ 18 years) treated at our tertiary university medical center in 2009–2019 according to the following criteria: (1) diagnosis of community-acquired bacterial meningitis, based on cerebrospinal fluid (CSF) examination with either (2a) a proven pathogen or (2b) ≥100 cells per microliter with ≥15% granulocytes, and (3) cerebral imaging available within 72 h after admission. Community-acquired meningitis was assumed in absence of a hospital stay and invasive spinal or cerebral procedures 8 weeks before diagnosis. Patient charts were reviewed and cerebral imaging was rated by an experienced neuroradiologist blinded to clinical outcome. The presence of early ischemic stroke and ECHO, defined as intracranial hemorrhage of any size, was rated on cerebral imaging up to 72 h after admission.

### Statistical Analysis

Standard descriptive statistics were used for all presented data. The neurological endpoint was functional outcome upon discharge measured by modified Rankin scale (mRS). Outcome was grouped as favorable (mRS 0–2), intermediate (mRS 3–4), and poor (mRS 5–6). Univariable and multivariable logistic regressions were performed to analyze the impact of early cerebrovascular complications and baseline characteristics on functional outcomes. Results are presented as odds ratios (OR) with 95% CI. The significance level was set to alpha = 0.05 and all analyses were two-sided. Statistical analyses were carried out with SPSS (version 25, IBM, NY).

## Results

### Patients and Baseline Characteristics

One hundred two adult patients with community-acquired meningitis treated at our institution between January 2009 and December 2019 were included. The median age was 57.6 years and 50% were female (Table 1). Upon presentation, the most common symptom was a headache in 69.5%, followed by neck stiffness in 65.9%, fever in 61.5%, and impaired consciousness in 51.5%. Any of these symptoms were present in 95.1% and focal neurological deficits were noted in 38.2%.

**Table 1:** Clinical baseline characteristics.

**Item**		**All patients (*n* = 102)**	**ECHO negative (*n* = 94)**	**ECHO positive (*n* = 8)**
Female, % (*n*)		50 (51)	47.9 (45)	75 (6)
Median age (IQR)		57.6 (45.8–71.4)	57.2 (45.8–71.4)	61.4 (45.2–75.2)
Causative pathogen, % (*n*)	*S. pneumoniae*	30.4 (31)	27.7 (26)	62.5 (5)
	*L. monocytogenes*	3.9 (4)	4.3 (4)	0
	*N. meningitidis*	2 (2)	2.1 (2)	0
	Other	39.2 (40)	41.5 (39)	12.5 (1)
	Unknown	24.5 (25)	24.4 (23)	25 (2)
Clinical signs, % (*n*)	Fever	61.5 (56)	55.3 (55)	50 (4)
	Headache	69.5 (66)	67 (63)	37.5 (3)
	Neck stiffness	65.9 (60)	60.6 (57)	37.5 (3)
	Impaired consciousness	51.5 (52)	25.8 (24)	62.5 (5)
	Focal neurological deficit	38.2 (39)	35.1 (33)	87.5 (7)
Infectious focus, % (*n*)	Mastoiditis/sinusitis/otitis	24.5 (25)	21.3 (20)	62.5 (5)
	Pneumonia	4.9 (5)	4.2 (4)	12.5 (1)
	Spinal empyema	3.9 (4)	4.2 (4)	0
	Endocarditis	2.9 (3)	3.2 (3)	0
	CSF leakage	2 (2)	2.2 (2)	0
	Vertebral discitis	2 (2)	2.2 (2)	0
	Other	10.7 (11)	11.7 (11)	0
	None detected	49 (50)	51.1 (48)	25 (2)
Antimicrobial treatment, % (*n*)		100 (102)	100 (94)	100 (8)
Dexamethasone treatment, % (*n*)		43.1 (44)	41.5 (39)	62.5 (5)
Pre-disposing condition, % (*n*)	Diabetes mellitus	12.7 (13)	12.7 (12)	12.5 (1)
	Immunosuppressive medication	6.9 (7)	7.4 (7)	0
	Active malignancy	3.9 (4)	4.3 (4)	0
	Alcoholism	3.9 (4)	4.3 (4)	0
	HIV	2.9 (3)	3.1 (3)	0
	None	69.6 (71)	68.1 (64)	87.5 (7)
CSF examination	Median lactate, mmol/l (IQR)	9.3 (4.6–14.1)	8.8 (4.5–13.6)	16.8 (7.1–20.5)
	Median glucose, mg/dl (IQR)	27 (5–53)	27 (6–53)	4 (0.5–37)
	Median protein, g/l (IQR)	2.4 (1.4–5.1)	2.4 (1.1–5.1)	2.7 (2.3–7.1)
	Median WBC, cells/μl (IQR)	1,220 (256–4,248)	1,047 (278–4,193)	2,730 (515–19,285)
	WBC <100/μl, % (*n*)	9.8 (10)	9.6 (9)	12.5 (1)
	WBC 100–1,000/μl, % (*n*)	36.3 (37)	38.7 (36)	12.5 (1)
	WBC > 1,000/μl, % (*n*)	52.9 (54)	51.6 (48)	75 (6)
	Protein >1–2 g/l, % (*n*)	17.6 (18)	20 (18)	0
	Protein > 2 g/l, % (*n*)	56.9 (58)	55.6 (50)	100 (8)

*CSF, cerebrospinal fluid; ECHO, early cerebral hemorrhage; IQR, interquartile range; WBC, white blood cell count; lyc, lymphocyte; gran, granulocyte*.

A predisposing immunosuppressive condition was present in 31.4%, and an infectious focus was detected in 51%. CSF examination was abnormal in all cases and revealed an increased white blood cell count with a median of 1,220 cells per microliter (IQR, 256–4,248). The median protein content was 2.4 grams per liter (IQR, 1.4–5.1). More details on CSF results are given in Table 1. Meningitis was caused most frequently by Streptococcus pneumoniae (30.4%), and no pathogen was found in 24.5%.

All patients received antimicrobial treatment. Dexamethasone was administered in 74.2% of Streptococcus pneumoniae infections (23 of 31) and 44.9% of all patients.

### ECHO-Positive Cases

**Figure 1:**
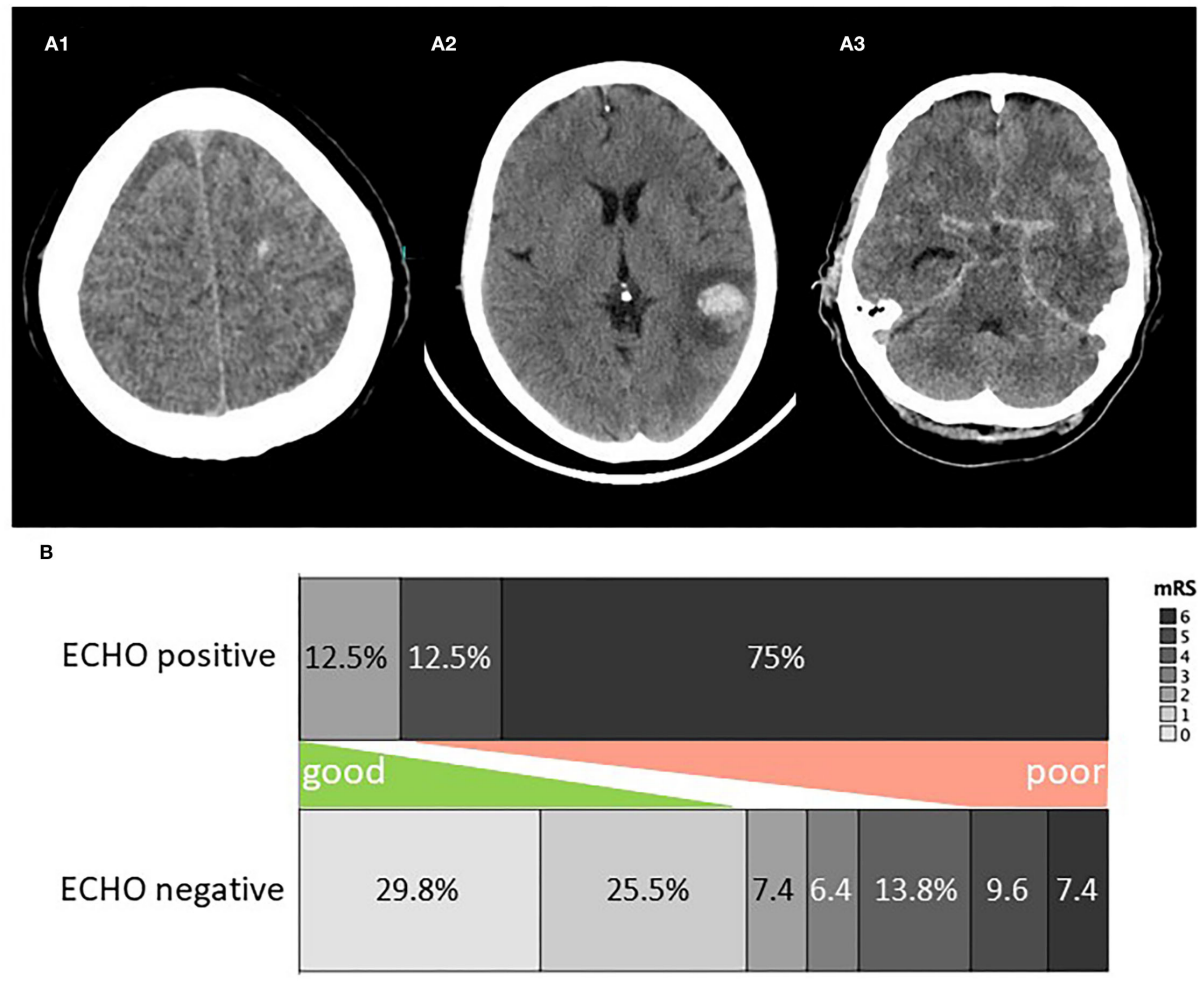
**(A)** Examples of ECHO on cerebral imaging. **(A1)** Microhemorrhage, **(A2)** lobar hemorrhage, **(A3)** subarachnoid hemorrhage. **(B)** Clinical outcome for ECHO positive and negative patients. ECHO, early cerebral hemorrhage; mRS, modified rankin scale score.

Cerebral imaging upon diagnosis was performed with computed tomography (CT) in 75.5%, magnetic resonance imaging (MRI) in 13.7%, and both in 10.8% of cases. 50% of patients received follow-up cerebral imaging within 72 h. 7.8% of patients were rated as ECHO-positive—among these, 50% on the first scan and 50% on the follow-up scan—, while ischemic stroke was detected in 15.7%. ECHO comprised intracerebral hematoma in 6 cases, microhemorrhage in 2 cases, and subarachnoid hemorrhage (in addition to intracerebral hematoma) in 1 case, all detected on CT (Figure 1).

Details on ECHO-positive patients are given in Table 1. One patient was anticoagulated before the diagnosis of ECHO (therapeutic low-molecular heparin). Suspected etiology or main risk factor for ECHO was sinus venous thrombosis (SVT) in 1 case, encephalitis and SVT in 2 cases, vasculitis in 1 case, subdural empyema drainage in 1 case, therapeutic anticoagulation in 1 case, and remained unknown in 2 cases.

### Clinical Outcome

CSF drainage was necessary for 6.9%. The median length of hospital stay was 19.5 days (IQR, 14.25–33). Clinical outcome upon discharge was favorable in 58.8%, intermediate in 18.6%, and poor in 22.5%. Figure 1B depicts the clinical outcome for patients with and without ECHO. Among patients who are ECHO-positive, the cause of death was sepsis, multiple ischemic strokes, hemorrhagic and ischemic stroke, and herniation due to brain edema in one case each, and withdrawal of care in 2 cases.

We performed logistic regression to analyze the impact of ECHO on poor clinical outcomes defined as mRS 5–6. The presence of ECHO or ischemic stroke on imaging, older age, reduced consciousness, and immunosuppression were predictive of poor outcomes in univariable analysis, while focal neurological deficit, CSF white blood cell count <1,000 per microliter, and the presence of mastoiditis, sinusitis or otitis as an infectious focus were not (Table 2). We conducted a multivariable logistic regression analysis considering significant predictors from the univariable analysis as mentioned above. The multivariable analysis identified the presence of ECHO [odds ratio (OR) 55.75, 95% CI: 3.08–1,008.48, *p* = 0.006], ischemic stroke (OR 15.06, 95% CI: 1.32–172, *p* = 0.029), older age (per 10 year increment: OR 2.56, 95% CI: 1.4–4.67; *p* = 0.002), and reduced consciousness (OR 4.21, 95% CI: 1.07–16.64, *p* = 0.04) as predictors of poor outcome (Table 2). Sensitivity analysis including only cases with proven pathogens confirmed these findings except for ischemic stroke, which was not significant (*p* = 0.065, Table 3).

**Table 2:** Logistic regression analysis of predictors for poor clinical outcome (mRS 5–6).

	**Univariable**			**Multivariable**		
	**OR**	**95% CI**	***p*-value**	**OR**	**95% CI**	***p*-value**
ECHO	34.13	3.92–296.86	0.001*	55.75	3.08–1008.48	0.006*
Ischemic stroke	6.62	1.68–26.05	0.007*	15.06	1.32–172.00	0.029*
Age (per 10 years)	1.68	1.19–2.39	0.003*	2.56	1.40–4.67	0.002*
Mastoid-/Ot-/Sinusitis	2.53	0.93–6.89	0.069			NI
Impaired consciousness	5.69	2.07–15.64	0.001*	4.21	1.07–16.64	0.040*
CSF WBC <1,000	0.95	0.37–2.44	0.91			NI
Focal deficit	1.05	0.41–2.72	0.92			NI
Immunosuppresssion	2.70	1.03–7.08	0.043*	2.62	0.71–9.76	0.15

*Asterisk indicates statistical significance. ECHO, early cerebral hemorrhage; CSF WBC, cerebrospinal fluid white blood cell count per microliter; NI, not included*.

**Table 3:** Logistic regression analysis of predictors for poor clinical outcome (mRS 5–6) among patients with culture-proven community-acquired bacterial meningitis (*n* = 76).

	**Univariable**			**Multivariable**		
	**OR**	**95% CI**	***p*-value**	**OR**	**95% CI**	***p*-value**
ECHO	23.79^+^	4.74–236.84	<0.001*	35.40^+^	5.19–492.2	<0.001*
Ischemic stroke	4.50	0.91–22.23	0.065			NI
Age (per 10 years)	1.65	1.09–2.39	0.016*	1.85^+^	1.21–3.10	0.003*
Mastoid-/Ot-/Sinusitis	2.05	0.70–6.02	0.19			NI
Impaired consciousness	5.27	1.74–15.98	0.003*	4.40^+^	1.35–15.33	0.014*
CSF WBC <1,000	1.00	0.35–2.83	1.00			NI
Focal deficit	0.56	0.19–1.60	0.28			NI
Immunosuppresssion	3.07	1.06–8.90	0.39*	2.93^+^	0.89–10.16	0.08

*Asterisk indicates statistical significance. ECHO, early cerebral hemorrhage; CSF WBC, cerebrospinal fluid white blood cell count per microliter; NI, not included. ^+^Due to complete separation, logistic regression including ECHO was calculated as Firth's penalized logistic regression*.

## Discussion

This retrospective study found evidence for the prognostic value of ECHO upon diagnosis or up to 72 h later in patients with community-acquired bacterial meningitis. ECHO, defined as any cerebral hemorrhagic lesion, was surprisingly frequent with 7.8% of patients, and the associated clinical outcome was deleterious with 75% mortality. In our cohort, it was the strongest predictor of poor clinical outcome, followed by ischemic stroke and reduced consciousness. While the poor outcome of a cerebral hemorrhage in meningitis is in line with former reports, the present study extends the prognostic value of ECHO to a defined and meaningful time frame early during disease (7–10).

Most patients with ECHO had pneumococcal meningitis, where cerebrovascular complications are especially common (8, 9). While histopathological studies demonstrated different mechanisms of brain injury in bacterial meningitis, including edema, necrotic lesions, and myelin loss, vascular damage is considered among the key contributors to brain damage (12). Accordingly, deceased patients showed high rates of hemorrhagic lesions in autopsy series (11, 12). Of note, it seems unlikely that ECHO itself was causative for poor outcomes in all cases reported here, as ECHO was composed of cerebral microhemorrhage in some patients, and larger ischemic lesions were less predictive. Therefore, the prognostic impact of ECHO suggests its utility as a clinical marker for more severe diseases.

In our cohort, most patients received CT, and all ECHOs were detected on CT. This is probably due to the low utilization of MRI in the reported cohort. While the fundamental principles of CT endure, modifications in data acquisition and reconstruction have enabled higher image quality (13). This might have contributed to the relatively high detection rate of small hemorrhagic lesions in this study. Of note, microhemorrhages attributable to cerebral amyloid angiopathy or chronic arterial hypertension were not detected, possibly because highly resolving susceptibility-weighted MRI sequences were rarely performed. Therefore, we propose not to encompass these known patterns as ECHO in future trials.

There are several limitations of this study. First, it is a single-center study at a German tertiary academic medical center, and there was considerable heterogeneity among patients, treatment, and type of meningitis, which limits generalization, and the number of patients with ECHO was small, limiting statistical power. As a retrospective study, the clinical outcome might be biased, and we only investigated patient outcomes upon discharge, but the high mortality among patients with ECHO renders the findings rather robust against this limitation. Furthermore, only patients with cerebral imaging and CSF analysis were included. Despite the known overuse of cerebral imaging in suspected meningitis, the former criterion might exclude patients with less severe disease (14), while the latter is expected to exclude patients with space-occupying lesions such as large intracerebral hemorrhage. Lastly, only half of the patients received follow-up CT within 72 h, as it was not performed routinely in patients who are clinically stable. Therefore, ECHO might be underdiagnosed in less severe cases, but the fact that 50% of ECHO were detected on the first scan attenuates this risk.

We conclude that the evaluation of ECHO might prove to be a valuable prognostic factor for clinicians confronted with decision-making in critically ill patients with community-acquired bacterial meningitis. Early identification of probable poor clinical outcomes is valuable for risk stratification and selection of more aggressive treatment regimens including early intracranial pressure targeted therapy (15), therapeutic hypothermia, or potential novel treatment strategies. Thus, early prognostication with ECHO should be further investigated in prospective trials.

## Data Availability Statement

The raw data supporting the conclusions of this article will be made available by the authors, without undue reservation.

## Ethics Statement

The studies involving human participants were reviewed and approved by Ethics Committee of the Rheinische Friedrich-Wilhelms University Bonn, Germany. Written informed consent for participation was not required for this study in accordance with the national legislation and the institutional requirements.

## Author Contributions

JW: conceptualization (equal), formal analysis (lead), investigation (lead), writing—original draft (lead), and writing—review, and editing (equal). JE: investigation (supporting), writing—review, and editing (equal). FL and AR: writing—review and editing (equal). TK: supervision (supporting), writing—review, editing (equal). JZ: conceptualization (equal), supervision (lead), writing—review, and editing (equal). All authors contributed to the article and approved the submitted version.

## Conflict of Interest

The authors declare that the research was conducted in the absence of any commercial or financial relationships that could be construed as a potential conflict of interest.

## Publisher's Note

All claims expressed in this article are solely those of the authors and do not necessarily represent those of their affiliated organizations, or those of the publisher, the editors and the reviewers. Any product that may be evaluated in this article, or claim that may be made by its manufacturer, is not guaranteed or endorsed by the publisher.
